# Supplementary material for: Neutrophil Activity and Extracellular Matrix Degradation: Drivers of Lung Tissue Destruction in Fatal COVID-19 Cases and Implications for Long COVID
Source: Biomolecules. 2024 Feb 17;14(2):236. doi: 10.3390/biom14020236 (PMC10886497; doi:10.3390/biom14020236)
Supplement: Supplementary file 1 [file biomolecules-14-00236-s001.zip › biomolecules-2831680-supplementary.pdf]

Figure S1

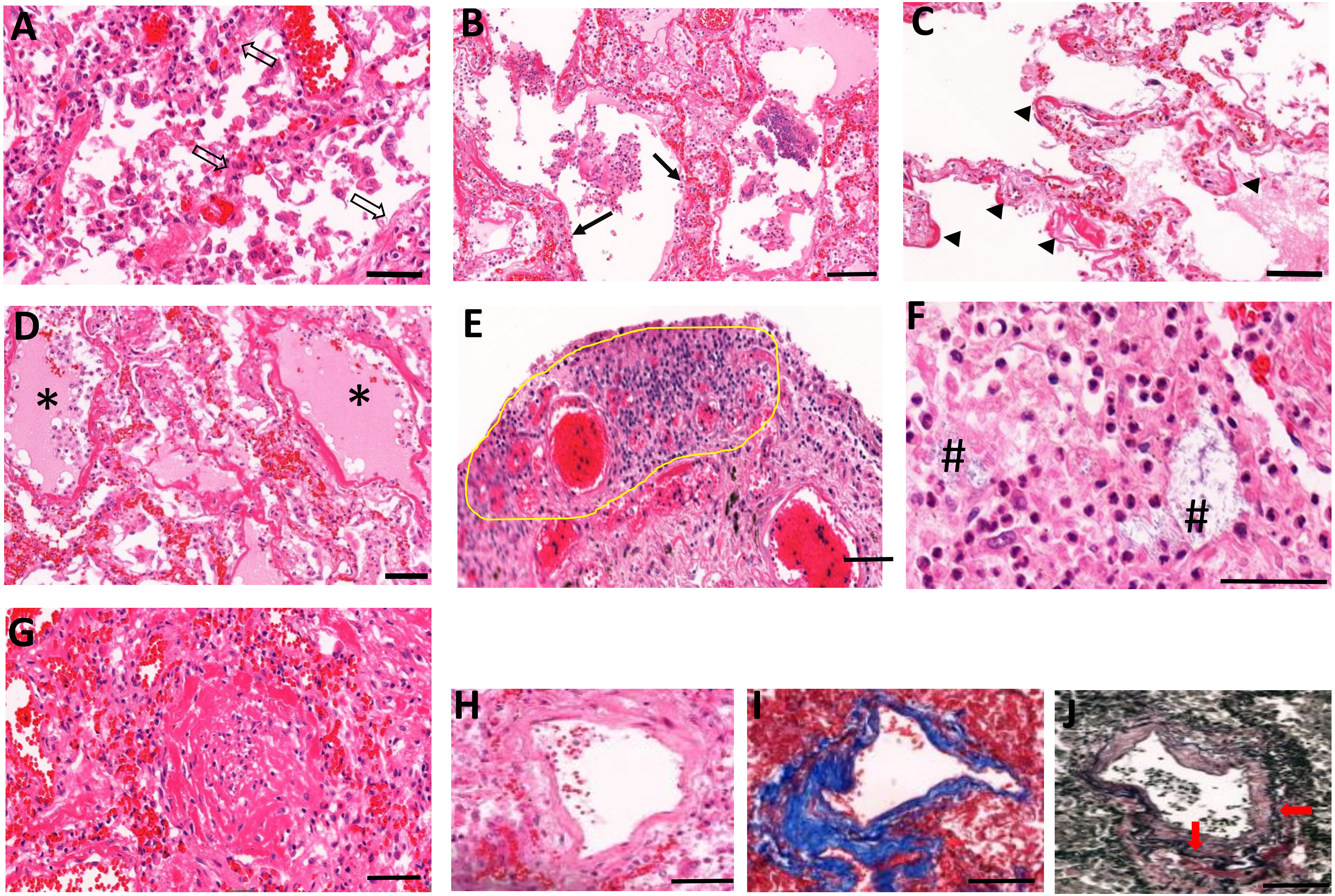

Supplementary Methods

Histopathology

Lung tissues were fixed in neutral-buffered formalin and embedded in paraffin. Lung sections were cut at 4 µm, dewaxed, rehydrated, and stained with hematoxylin and eosin. Various lung pathologic features including acute or organizing diffuse alveolar damage (DAD), alveolar hemorrhage, thrombosis, and fibrotic remodeling described earlier in COVID-19 patients[1-3] were evaluated by an experienced pathologist (AK) in a blinded manner. Pathological lesions of acute DAD were characterized by disrupted alveolar epithelium, epithelial sloughing, formation of hyaline membranes, necrotizing bronchiolitis, interstitial inflammation, oedema, fibrin deposition, vasculitis, pulmonary hemorrhage, and microvascular thrombosis. The organizing form of DAD was defined as loosely organized fibrosis, interstitial fibrotic changes, and abnormal remodeling of epithelium with hyperplasic type II pneumocytes and proliferative airway epithelium. Fibrotic changes were defined as extensive and dense interstitial fibrosis replacing lung parenchyma and disintegrating alveolar architecture. The histopathological grading was 0-absent, 1-mild, 2-moderate, and 3-severe. The lung sections were stained with Verhoff van Gieson (VVG) and orcein for detecting elastin fibers and Masson's trichrome for identification of collagen depositions.

Immunohistochemistry

Lung tissue sections were subjected to immunohistochemistry analysis using Bond™ Polymer Refine Red Detection Kit (Leica Biosystems, IL) with primary antibodies for the detection of neutrophil granule proteins including NE (1:400 dilution), myeloperoxidase (MPO, 1:10000 dilution); alveolar type II epithelium marker, TTF-1, (1:1 dilution); and peptidylarginine

deiminase 4 (PAD4, 1:300 dilution), a marker for neutrophil extracellular traps (NETs) induction. Antigen epitope retrieval was conducted with EnVision FLEX Target Retrieval Solution High pH (K800421-2, Dako/Agilent, USA) and low pH IHC Antigen Retrieval Solution (00-4955-58, Invitrogen, USA) using a Biocare Decloaking Chamber (Biocare Medical, USA) for 5 minutes at 120 °C. Following antigen retrieval, tissue sections were incubated with primary antibodies as described above, followed by Horse radish peroxidase-conjugated secondary antibodies. Color was developed using universal diaminobenzidine chromogen substrate. Whole slide images were captured using CaseViewer 3DHITECH Ltd. software.

Elastin fiber morphometry

Elastin fibers in the alveolar walls were detected by VVG and orcein staining and images were captured as described above. Lengths of the elastin fibers were measured using ImageJ software analysis (National Institute of Health, April, 2019; ImageJ with 64-bit Java 1.8.0_172). Images from 10 randomly selected fields at 400x were captured. The images were first normalized for scale, the actual lengths of the elastin fibers were measured in micrometers (μm) in a blinded manner, and the curvatures of the elastin fibers along the walls of the alveoli were evaluated using freehand length measurement.[4] The bundle of elastin fibers was measured as a single fiber unless the fibers were distinctly separated from each other. Elastin fibers often appear as single detached, fragmented, or degraded fibers in COVID-19 patients. These fibers were counted separately to measure their lengths. All data were exported into an Excel file and the average fiber length in μm was determined. Each fiber was counted only once, and no extra weighting was used for larger/wider fibers. Elastin fibers staining in the airways or surrounding bronchioles, or pulmonary blood vessels were excluded.

Quantification of collagen and measurement of collagen:elastin ratio in the lungs of COVID-19 patients.

Collagen in the lung sections was detected using Masson's Trichrome stain and lung sections were imaged as described above, and images were analyzed by ImageJ software. For the measurement of collagen density, 10 random fields were selected from each lung section. The collagen staining was processed by selecting Masson's Trichrome specific staining using color deconvolution plugin software in ImageJ that separates red, blue, and green components from the image. Thresholds for collagen staining were established for each slide by enhancing the contrast to a point at which the collagen staining was easily identified by blue staining and the density of the collagen was measured over total lung area from the image.[45] The collagen was measured as percent collagen density in total lung area of the image. No weighting was performed for the intensity of blue staining. Further, adjacent serial lung sections were stained with Masson's Trichrome (collagen) and orcein (elastin) for quantitative measurement of collagen:elastin ratio as a fold change after both stains were normalized to the total lung area in the image captured in the same areas of the sections.

Protein extraction from formalin-fixed paraffin-embedded (FFPE) lung tissues and western blot analysis:

FFPE lung tissues (10 µm sections) from non-COVID-19 and COVID-19 patients were transferred into 1.5 ml centrifuge tubes, dewaxed in xylene for 10 min and centrifuged. T issue pellets were washed 3x with xylene and rehydrated with graded concentrations of ethanol (100 %, 85 % and 75 % for 2 min in each). After the ethanol wash, proteins in the tissue pellets were

solubilized in Tris-SDS buffer, pH 8.0, by homogenization and sonication. Tissue homogenates were centrifuged at 10,000 g for 20 min and the supernatants were measured for total protein concentrations. Equal amounts of proteins were resolved by 12% sodium dodecyl sulfate-polyacrylamide gel electrophoresis, followed by transfer onto nitrocellulose membrane. Membranes were then blocked with 5% bovine serum albumin and incubated with anti-elastin (1:1000 dilution) and anti-histone H3 Pan antibodies (loading control, 1:4000 dilution) overnight at 4 ^{0}C. Next day, membranes were washed in TBST and incubated with IRDye 800CW and IRDye 600CW conjugated secondary antibodies. The membranes were washed 3 times and signal was measured using the Odyssey Infrared Imaging System (LI-COR Biosciences).

Colorimetric determination of citrulline residues in proteins:

To measure the total citrullinated proteins in the lung homogenates, an equal amount of proteins from non-COVID-19 and COVID-19 patients were added to the chromogenic reagent (Diacetyl-monoxime Solution + Antipyrine Solution + Acid-Ferric Solution, at 1:2:3), mixed well, and the tubes covered with aluminum foil. For standard, 0.5 to 10 nmoles of citrulline were prepared as described above. The samples and standards were heated for 25 min in a boiling water bath and cooled to RT on ice. The color developed was read at 464 nm.[6] Quantification of the citrullinated proteins in the lung homogenates was determined using the citrulline-protein standard curve.

ELISA for evaluation of the NE-A1AT complexes in the plasma samples:

Standard NE-A1AT complexes were prepared by combining 2.5 μM NE with 10 μM A1AT in 100ul of PBS, and incubated at 37^{0}C for 30 and 60 min. After the incubation, aliquots were snap frozen and kept at -80^{0}C until further use. For ELISA, 96-well plates were coated with the anti-

NE antibody at 1 μg/ml and incubated overnight at 4^0C. On the following day, the plates were washed once with PBS Tween-20, and blocked with 1 % BSA in PBS for 1 hour at RT. The plates were then incubated with plasma samples collected from non-COVID-19 or COVID-19 patients for 2 hours at RT, followed by 3 washes, and further incubation with rabbit polyclonal anti-A1AT antibody at 1:10000 dilutions for 1 hour. Next, the plates were washed 3 times, and incubated with anti-rabbit-IgG-AP for 1 hour at 1:2000, followed by 3 washes, and further incubation with para-Nitrophenylphosphate substrate for 30min at RT in the dark. The color developed was read at 405 nm.[7-8] The NE-A1AT known standard complexes were used to quantitate complexes present in the COVID-19 and non-COVID-19 plasma samples.

References

1. Mauad T, Duarte-Neto AN, da Silva LFF, de Oliveira EP, de Brito JM, do Nascimento ECT, de Almeida Monteiro RA, Ferreira JC, de Carvalho CRR, do Nascimento Saldiva PH, Dolhnikoff M. Tracking the time course of pathological patterns of lung injury in severe COVID-19. Respir Res. 2021;22:32.
2. Merdji H, Mayeur S, Schenck M, Oulehri W, Clere-Jehl R, Cunat S, Herbrecht JE, Janssen-Langenstein R, Nicolae A, Helms J, Meziani F, Chenard MP; CRICS TRIGGERSEP Group (Clinical Research in Intensive Care, Sepsis Trial Group for Global Evaluation, Research in Sepsis). Histopathological features in fatal COVID-19 acute respiratory distress syndrome. Med Intensiva (Engl Ed). 2021;45:261-270.
3. Valdebenito S, Bessis S, Annane D, Lorin de la Grandmaison G, Cramer-Bordé E, Prideaux B, Eugenin EA, Bomsel M. COVID-19 Lung Pathogenesis in SARS-CoV-2 Autopsy Cases. Front Immunol. 2021;12:735922.
4. Zweers MC, van Vlijmen-Willems IM, van Kuppevelt TH, Mecham RP, Steijlen PM, Bristow J, Schalkwijk J. Deficiency of tenascin-X causes abnormalities in dermal elastic fiber morphology. J Invest Dermatol. 2004;122:885-91.
5. Kottmann RM, Sharp J, Owens K, Salzman P, Xiao GQ, Phipps RP, Sime PJ, Brown EB, Perry SW. Second harmonic generation microscopy reveals altered collagen microstructure in usual interstitial pneumonia versus healthy lung. Respir Res. 2015;16:61.

6. Dwivedi N, Hedberg A, Zheng YY, Neeli I, Satoh M, Morel L, Rekvig OP, Radic M. B Cell Tolerance to Deiminated Histones in BALB/c, C57BL/6, and Autoimmune-Prone Mouse Strains. Front Immunol. 2017;8:362.

7. Neumann S, Hennrich N, Gunzer G, Lang H. Enzyme-linked immunoassay for human granulocyte elastase in complex with alpha 1-proteinase inhibitor. Adv Exp Med Biol. 1984;167:379-90

8. Engelmaier A, Weber A. Sensitive and specific measurement of alpha$_1$-antitrypsin activity with an elastase complex formation immunosorbent assay (ECFISA). J Pharm Biomed Anal. 2022;209:114476
